# Supplementary material for: Smooth Muscle Myosin Localizes at the Leading Edge and Regulates the Redistribution of Actin-regulatory Proteins during Migration
Source: Cells. 2022 Jul 29;11(15):2334. doi: 10.3390/cells11152334 (PMC9367404; doi:10.3390/cells11152334)

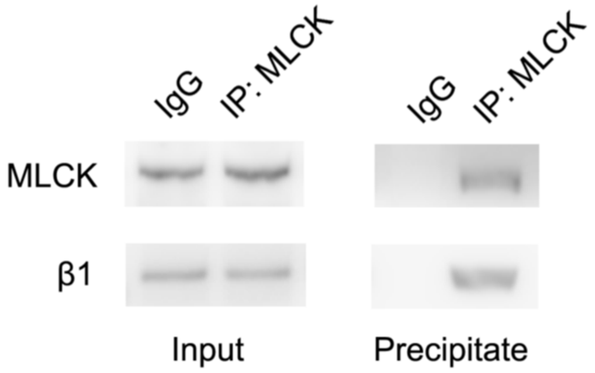


**Figure S1**. **Interaction of integrin β1 with MLCK.** Input and MLCK immunoprecipitates of human airway smooth muscle cells were assessed by immunoblotting. Integrin β1 is found in MLCK immunoprecipitates. Blots are representative of three identical experiments.

Original blots for Figure 2A

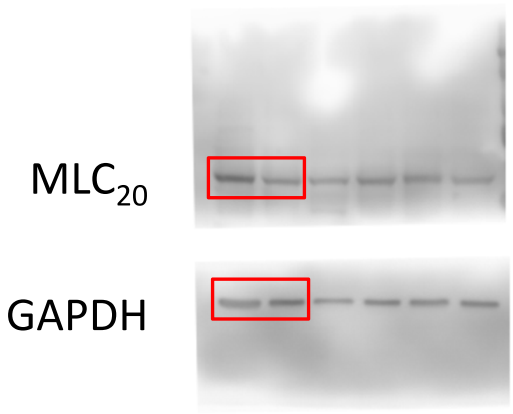


Original blots for Figure 2C

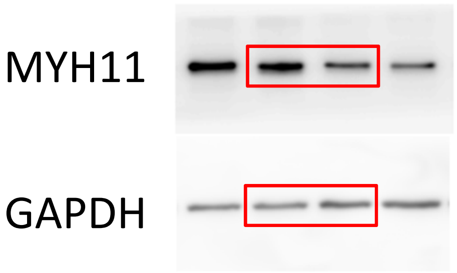

Original blots for Figure 6A

β1

GAPDH

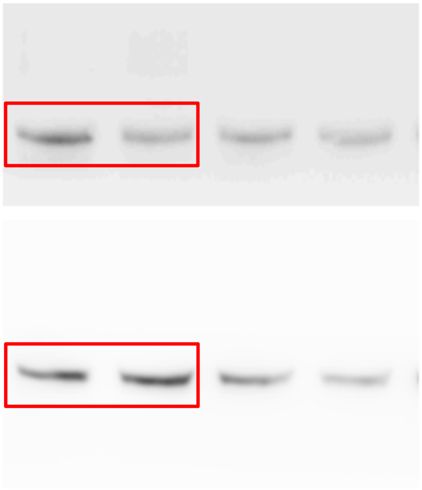

Supplement: Supplementary file 1 [file cells-11-02334-s001.zip › cells-1838183-supplementary.pdf]
